# Comparative effects of different magnetic stimulation modalities on upper limb motor function and activities of daily living in patients with stroke: a systematic review and network meta-analysis

**DOI:** 10.3389/fneur.2026.1842774

**Published:** 2026-10-27

**Authors:** Shuijin Yang, Yan Rao, Chen Wei, Shuqi Jia, Qiang Liu

**Affiliations:** 1 Hunan Institute of Engineering, Xiangtan, China; 2 Shanghai Normal University Tianhua College, Shanghai, China; 3 Shenyang Normal University, Shenyang, China; 4 Shanghai University of Sport, Shanghai, China; 5 Shanghai Lixin University of Accounting and Finance, Shanghai, China

**Keywords:** activities of daily living, magnetic stimulation, network meta-analysis, stroke, upper limb motor function

## Abstract

**Objective:**

To systematically evaluate the effects of different magnetic stimulation (MS) modalities on upper limb motor function and activities of daily living (ADL) in patients with stroke through a network meta-analysis.

**Methods:**

Embase, Web of Science, PubMed, Wanfang, VIP, and CNKI were searched from inception to January 2026 for randomized controlled trials evaluating MS interventions for upper limb motor function and activities of daily living (ADL) in patients with stroke. A frequentist network meta-analysis was performed using standardized mean difference (SMD) with 95% confidence interval (CI). The surface under the cumulative ranking curve (SUCRA) was used to estimate the relative ranking probabilities of interventions. Risk of bias was assessed using RoB 2, and the certainty of evidence was evaluated using GRADE.

**Results:**

A total of 48 randomized controlled trials were included. For upper limb motor function, rPMS (SMD = 0.65, 95% CI: 0.19 to 1.12), rTMS (SMD = 0.96, 95% CI: 0.68 to 1.24), and combined MS (SMD = 1.01, 95% CI: 0.47 to 1.55) were all superior to conventional rehabilitation. SUCRA rankings suggested that combined MS ranked highest, followed by rTMS. However, pairwise comparisons among active magnetic stimulation interventions did not reach statistical significance. For ADL, rPMS (SMD = 0.60, 95% CI: 0.26 to 0.95), rTMS (SMD = 0.76, 95% CI: 0.56 to 0.97), and combined MS (SMD = 1.27, 95% CI: 0.94 to 1.59) significantly improved ADL compared with conventional rehabilitation. Combined MS was also superior to rPMS and rTMS alone. Sensitivity analysis excluding studies that reported only the Barthel Index showed broadly consistent results. No clear evidence of publication bias or small-study effects was detected. The certainty of evidence was rated as low.

**Conclusion:**

MS interventions may improve upper limb motor function and ADL in patients with stroke. Combined MS and rTMS showed relatively high ranking probabilities for upper limb motor recovery, whereas combined MS showed the highest ranking probability for ADL improvement. Given the heterogeneity among included studies, non-significant differences in some direct comparisons, and low certainty of evidence, these findings should be interpreted cautiously and confirmed by high-quality head-to-head trials.

**Systematic review registration:**

https://www.crd.york.ac.uk/PROSPERO/view/CRD420261302905, Identifier: CRD420261302905.

## 1 Introduction

Stroke refers to an acute neurological deficit caused by cerebrovascular circulatory disturbance. It is characterized by high incidence, mortality, disability, and recurrence rates, and ranks as the leading cause of death among Chinese residents (1). Stroke therefore represents a major public health challenge worldwide (2). Projections suggest that, if current trends continue, the burden of stroke in China will continue to increase by 2050 and stroke will remain a leading cause of death and disability in the Chinese population (3). The latest global forecasting model published suggests that annual stroke-related deaths may rise from 6.6 million in 2020 to 9.7 million by 2050 (4). Meanwhile, the disability burden among stroke survivors is also increasing (5). Among the various sequelae, upper limb motor dysfunction is one of the most common complications after stroke (6), affecting approximately 80% of patients (7). Previous studies have demonstrated a close positive association between upper limb motor control and activities of daily living (ADL), which substantially affects patients’ independence and quality of life (8, 9). Therefore, exploring and implementing systematic and standardized rehabilitation strategies is of great clinical importance for improving the prognosis of patients with stroke.

Conventional physical therapy often faces challenges such as a long recovery period and therapeutic plateau (10), and may not fully meet patients’ functional recovery needs. In recent years, with advances in neuroscience, magnetic stimulation (MS) techniques based on the principle of neuroplasticity have emerged as promising non-invasive neuromodulation approaches and have shown therapeutic potential in upper limb rehabilitation after stroke. The MS techniques commonly used in clinical practice mainly include repetitive transcranial magnetic stimulation (rTMS), which acts on the central nervous system, and repetitive peripheral magnetic stimulation (rPMS), which acts on the peripheral nervous system (11). rTMS induces an electric field through a magnetic field, thereby modulating cortical excitability (12) and influencing cerebral metabolic processes and neuronal activity. rTMS can induce synchronized neuronal discharges over large-scale networks and has relatively high spatial resolution, allowing more precise stimulation of target brain regions. In contrast, rPMS is a non-invasive technique in which the MS coil is positioned over the patient’s peripheral limbs or trunk. The magnetic field can penetrate the skin and soft tissues and act directly on excitable structures in peripheral nerve-muscle tissues, thereby modulating neural and/or muscular excitation or inhibition and promoting proprioceptive recovery and muscle function reconstruction (13, 14). Combined MS generally refers to a composite intervention in which central rTMS and peripheral rPMS are applied simultaneously or sequentially within the same treatment period. This approach aims to promote post-stroke motor recovery through the synergistic effects of central cortical excitability modulation and enhanced peripheral sensorimotor input.

Although previous systematic reviews and meta-analyses have supported the application of MS in post-stroke upper limb rehabilitation, their conclusions have not been entirely consistent. The efficacy of rTMS may be influenced by stimulation frequency, intensity, number of treatment sessions, and total number of pulses (15). Although rPMS may improve upper limb motor function and activity limitation, uncertainty remains regarding its effect size and the populations most likely to benefit (16). In addition, previous evidence suggests that rPMS alone or combined with rTMS may improve upper limb function and activity independence, but combined treatment is not necessarily superior to rTMS alone (14). These discrepancies may be related to differences in stroke stage, stroke type, stimulation parameters, treatment dose, stimulation target, concurrent rehabilitation programs, and outcome measures. However, existing evidence has mainly focused on comparisons of a single technique (rTMS or rPMS) with conventional therapy (17), while systematic direct comparisons and comprehensive evaluations of the relative efficacy of different MS modalities and their combined protocols, such as rTMS combined with rPMS, remain limited. Therefore, this study aimed to systematically synthesize evidence from relevant randomized controlled trials using network meta-analysis, and to directly and indirectly compare the effects of rTMS, rPMS, combined MS, and other MS interventions on upper limb motor function and ADL in patients with stroke. Through quantitative analysis, this study sought to evaluate the relative effects and ranking trends of different MS protocols, with the aim of providing evidence-based support for clinical rehabilitation decision-making and informing future research priorities.

## 2 Methods

This study was conducted in accordance with the PRISMA reporting guidelines (18) and was registered in the International Prospective Register of Systematic Reviews (PROSPERO; No. CRD420261302905). Following registration, the protocol was amended in response to reviewers’ comments to update the data synthesis methods and adopt the Cochrane RoB 2 tool for risk-of-bias assessment. These amendments aimed to strengthen methodological rigor and support interpretation of the findings. All analyses specified in the amended protocol were completed.

### 2.1 Search strategy

Two reviewers independently searched Embase, Web of Science, PubMed, Wanfang Database, VIP Database, and China National Knowledge Infrastructure (CNKI) to identify randomized controlled trials (RCTs) investigating the effects of magnetic stimulation on upper limb motor function and ADL in patients with stroke. The search period extended from database inception to January 2026. In addition, the reference lists of the included studies were manually searched. The detailed search strategy is provided in Supplementary Material 1.

### 2.2 Inclusion and exclusion criteria

The inclusion criteria were as follows: (1) participants were aged ≥18 years, had a first-ever stroke, and were diagnosed by computed tomography (CT) or magnetic resonance imaging (MRI); (2) the intervention in the experimental group included one of the following magnetic stimulation modalities or their combination: rTMS, applied to the central motor cortex; rPMS, applied to the affected upper limb muscles, nerve trunks, or joints to promote functional recovery by inducing muscle contraction or proprioceptive input; or combined MS, defined as rTMS combined with rPMS; (3) the control group received conventional rehabilitation therapy or sham stimulation; (4) the main outcomes included upper limb motor function or ADL; (5) the study design was an RCT; and (6) the publication language was Chinese or English.

The exclusion criteria were as follows: (1) duplicate publications; (2) studies from which data could not be calculated or extracted; (3) studies using specific stimulation protocols that were outside the scope of this review, namely theta burst stimulation (TBS), including intermittent TBS (iTBS) and continuous TBS (cTBS); (4) studies with ineligible interventions or outcome measures; and (5) studies for which the full text was unavailable.

### 2.3 Study selection and data extraction

After the retrieved records were imported into EndNote and duplicates were removed, two reviewers independently screened the literature and extracted data. The extracted data were entered into a predesigned form and double-checked for accuracy. Any disagreement was resolved through discussion with a third reviewer. The extracted information included the first author, year of publication, baseline characteristics of participants (age, sex, disease duration, stroke type, etc.), intervention characteristics (type of magnetic stimulation, stimulation site, stimulation frequency, intensity, single-session duration, total treatment duration, and concomitant rehabilitation), and outcome measures.

The upper extremity section of the Fugl-Meyer Assessment (FMA-UE) was selected as the primary measure of motor function. FMA-UE is internationally recognized as a widely used and well-validated measure for assessing post-stroke motor recovery (19) and has good reliability, validity, and sensitivity. Previous studies have shown a high level of agreement between the Barthel Index (BI) and the modified Barthel Index (MBI) in the assessment of patients with stroke, with a Pearson correlation coefficient exceeding 0.90 (20). Because the MBI represents a refinement and sensitivity improvement of the BI scoring system, the two scales substantially overlap in their assessment dimensions and clinical interpretation of ADL (21). Therefore, this study considered BI and MBI to have acceptable psychometric comparability and combined them as the ADL outcome. Considering potential differences in scoring ranges and sensitivity, the standardized mean difference (SMD) was used as the effect size to reduce the influence of differences in scale scoring on the pooled analysis. For each outcome, pre-post change scores were extracted and analyzed, with the change score defined as the post-intervention score minus the baseline score. To avoid including multiple comparisons from studies with more than one repetitive transcranial magnetic stimulation group in the meta-analysis, relevant groups were combined according to the recommendations of the Cochrane Handbook for Systematic Reviews of Interventions (22). When the original studies reported only medians, interquartile ranges, or ranges, the optimized estimation formulas proposed by Wan et al. and Luo et al. (23, 24).

### 2.4 Quality assessment

The methodological quality of the included randomized controlled trials was assessed using the Risk of Bias 2 (RoB 2) tool across five domains: the randomization process, deviations from intended interventions, missing outcome data, measurement of the outcome, and selection of the reported result (25). The risk of bias in each domain was judged as “low risk of bias,” “some concerns,” or “high risk of bias.” The overall risk of bias for each study was determined based on the domain-level assessments. In addition, the Grading of Recommendations Assessment, Development and Evaluation (GRADE) approach was used to assess the certainty of evidence for the outcomes (26). The certainty of evidence was classified into four levels: high, moderate, low, and very low. The GRADE assessment considered risk of bias, inconsistency, indirectness, imprecision, and publication bias. Two reviewers independently performed the assessments, and any disagreement was resolved through discussion with a third reviewer until consensus was reached.

### 2.5 Statistical analysis

Because the included studies of MS differed in stimulation parameters, including stimulation site, intensity, frequency, and treatment duration, and because all outcomes were continuous variables, the standardized mean difference (SMD) with 95% confidence interval (CI) was used as the effect size. The direction of effect was defined such that an increase in score indicated functional improvement; therefore, an SMD > 0 indicated that the corresponding magnetic stimulation intervention was superior to the control or comparator intervention. Network meta-analysis was performed using Stata, and network evidence plots were generated. The network structure and connectivity were first assessed, and network meta-analysis was conducted only when the network was connected. Before interpreting indirect comparisons, the transitivity assumption was assessed clinically by comparing the distribution of potential effect modifiers across treatment comparisons, including stroke stage, stroke type, disease duration or baseline severity where reported, stimulation frequency and intensity, stimulation site, treatment dose or duration, and concomitant rehabilitation. These characteristics were extracted from the included studies and summarized in Table 1 and Supplementary Material 2. Because the included comparisons were based on adult patients with stroke, clinically comparable magnetic stimulation modalities, and commonly used post-stroke motor function and ADL outcomes, indirect comparisons were considered clinically reasonable; however, residual differences in effect modifiers were considered when interpreting treatment rankings. Heterogeneity was evaluated using τ^2^, Cochran’s Q test, and I^2^. A random-effects model was used because statistical and clinical heterogeneity were present. In networks containing both direct and indirect evidence, the consistency assumption was assessed using global inconsistency tests and local node-splitting methods. When *p* > 0.05, no significant inconsistency was considered to have been detected, and a consistency model was used for analysis. When *p* ≤ 0.05, possible inconsistency was considered and interpreted together with inconsistency models and node-splitting results. SUCRA values were used to rank different rehabilitation strategies. Larger SUCRA values indicated higher ranking probabilities, but ranking results were interpreted together with relative effect estimates and their 95% CIs. Publication bias and small-study effects were assessed using comparison-adjusted funnel plots and Egger regression tests. For the ADL outcome, studies reporting only BI were excluded and only studies using MBI were retained for sensitivity analysis to assess the robustness of combining BI and MBI. All statistical analyses and figure generation were performed using Stata 17.

**Table 1:** Basic characteristics of included studies.

Author	Sample size	Age (T/C)	Disease duration (T/C)	Stroke type	Intervention (T/C)		Outcome
Bao 2021 (27)	T1:40 C:40	T1:57.5 ± 1.5 C:57.8 ± 1.6	≤15d	Ischemic	rTMS	Sham stimulation +Conventional rehab	①
	T2:40	T2:57.6 ± 1.3					
Chen BM 2023 (28)	T:15 C:15	T:59.8 ± 8.29 C:55.6 ± 7.11	T:29.87 ± 20.59d C:32.53 ± 21.56d	Both	rTMS	Sham stimulation +Conventional rehab	①
Chen 2024 (29)	T:29 C:30	T:55.17 ± 13.51 C:64.75 ± 9.09	T:26.83 ± 7.51d C:23.75 ± 5.55d	Both	rTMS	Conventional rehab	①
Chen J 2025 (30)	T:16 C:16	T:62.31 ± 10.47 C:60.31 ± 9.67	T:36.60 ± 30.07d C:50.95 ± 54.45d	Both	rPMS	Conventional rehab	①②
Chen 2021 (31)	T:30 C:30	T:64.13 ± 13.20 C:61.37 ± 11.90	T:2.00 ± 1.34 m C:2.17 ± 1.11 m	Both	rTMS	Sham stimulation + Conventional rehab	①②
Chen YY 2023 (32)	T1:22	T1:62.18 ± 10.24	T1:7.23 ± 0.66	Ischemic	rTMS + rPMS	Conventional rehab	①②
	T2:20	T2:59.55 ± 8.86	T2:8.93 ± 4.01		rTMS		
Chen 2018 (33)	T:91 C:89	T:56.89 ± 12.43 C:57.53 ± 13.05	T:23.19 ± 7.41d C:24.06 ± 8.17d	Both	rTMS	Conventional rehab	①②
Cheng 2025 (34)	T1:17	T1:52.82 ± 14.45	T1:1.81 ± 1.15 m	Both	rTMS + rPMS	Conventional rehab	①②
	T2:17	T2:58.29 ± 14.71	T2:2.10 ± 1.46 m		rTMS + Sham rPMS		
Fu 2024 (35)	T1:20 C:20	T1:60.4 ± 10.7 C:62.8 ± 11.7	T1:43.7 ± 24.3d C:41.2 ± 25.2d	Ischemic	rTMS	Sham stimulation + Conventional rehab	①②
	T2:20	T2:62.6 ± 9.9	T2:44.3 ± 23.6d		rTMS		
Hu 2025 (36)	T:30 C:29	T:58.83 ± 10.71 C:60.15 ± 8.33	T:47.30 ± 25.24d C:45.86 ± 23.01d	Both	rPMS	Conventional rehab	①②
Li 2016 (37)	T1:20 C:20	T1:50.87 ± 6.14 C:51.42 ± 6.56	T1:2.43 ± 1.53 m C:2.50 ± 1.56 m	Ischemic	rTMS	Conventional rehab	①②
	T2:20	T2:51.52 ± 7.08	T2:2.38 ± 1.69 m		rTMS	Conventional rehab	
Liang 2018 (38)	T:39 C:39	T:64.3 ± 11.8 C:64.7 ± 10.6	T:18.3 ± 11.5d C:19.4 ± 9.6d	Both	rTMS	Sham stimulation + Conventional rehab	①②
Liu 2019 (39)	T:20 C:20	T:61.35 ± 9.43 C:55.00 ± 11.86	T:2.81 ± 1.27 m C:3.11 ± 1.37 m	Ischemic	rTMS	Conventional rehab	①②
Liu XM 2018 (40)	T:50 C:50	T:55.0 ± 12.4 C:55.6 ± 10.3	T:66.3 ± 20.5d C:65.6 ± 20.4d	Both	rTMS	Conventional rehab	①②
Liu Y 2018 (41)	T:13 C:10	T:56.90 ± 9.02 C:55.38 ± 8.40	T:4.50 ± 1.90 m C:4.85 ± 2.08 m	Ischemic	rTMS	Conventional rehab	①②
Luo 2024 (42)	T1:13	T1:55.80 ± 14.09	T1:25.20 ± 6.74d	Hemorrhagic	rTMS	Conventional rehab	①②
	T2:13	T2:53.60 ± 16.45	T2:25.60 ± 7.38d		rPMS		
	T3:14	T3:56.40 ± 13.83	T3:29.50 ± 7.93d		rTMS + rPMS		
Peng 2024 (43)	T1:15 C:15	T1:56.6 ± 12.1 C:59.4 ± 18.7	T1:19.0 ± 2.9d C:18.8 ± 3.1d	Ischemic	rTMS	Conventional rehab	①
	T2:15	T2:54.8 ± 10.6	T2:18.5 ± 2.9d				
	T3:15	T3:58.2 ± 13.1	T3:19.6 ± 2.6d				
Sun 2025 (44)	T1:20 C:19	57.30 ± 8.43/58.70 ± 16.24	1.51 ± 1.82 m/1.48 ± 1.46 m	Both	rTMS	Conventional rehab	①②
	T2:19	57.50 ± 17.14	1.09 ± 0.93 m				
Xia 2022 (45)	T1:40 C:40	T1:70.1 ± 1.7/C:69.9 ± 1.8	T1:79.1 ± 37.6d/C:72.3 ± 34.8d	Ischemic	rTMS + rPMS	Conventional rehab	①②
	T2:40	T2:69.3 ± 1.9	T2:76.6 ± 39.3d		rTMS		
Xiao 2019 (46)	T1:20 C:19	60.4 ± 11.77/57.68 ± 10.66	64.50 ± 35.19d/72.95 ± 41.38d	Ischemic	rTMS	Sham stimulation + Conventional rehab	①②
	T2:21	58.71 ± 9.97	68.62 ± 26.63d				
Xie 2024 (47)	T1:19 C:20	T1:61.6 ± 8.3/64.0 ± 9.9	33.05 ± 14.19d/30.25 ± 13.47d	Both	rPMS	Conventional rehab	①②
	T2:19	T2:62.6 ± 10.2	37.79 ± 15.16d				
Yan 2021 (48)	T1:18	T1:60.56 ± 8.75	32.75 ± 18.55d	Both	rTMS + rPMS	Conventional rehab	①②
	T2:17	T2:58.29 ± 17.25	43.38 ± 24.76d		rTMS + Sham rPMS		
Yu 2021 (49)	T:13 C:14	T:55.85 ± 6.71/55.57 ± 9.43	85.08 ± 50.41d/81.86 ± 47.99d	Both	rPMS	Conventional rehab	①②
Yuan 2025 (50)	T:20 C:20	T:52.50 ± 10.94/56.15 ± 10.95	71.90 ± 53.50d/74.50 ± 46.75d	Both	rPMS	Conventional rehab	①②
Zhao 2015 (51)	T:20 C:20	T:57.7 ± 9.5/55.5 ± 13.2	54.1 ± 14.3d/52.1 ± 13.8d	Ischemic	rTMS	Conventional rehab	①
Zhao 2018 (52)	T:36 C:39	T:54.0 ± 11.4/C:56.2 ± 12.7	4.0 ± 2.0w/4.3 ± 3.1w	Both	rTMS	Conventional rehab	①
Zhou 2020 (53)	T:30 C:28	T:59.73 ± 10.41/C:61.75 ± 11.43	10.70 ± 7.57d/10.50 ± 7.38d	Both	rTMS	Conventional rehab	①②
Aşkın 2017 (54)	T:20 C:20	T:56.75 ± 11.46/C:58.80 ± 12.02	T:28.35 ± 15.34d/C:24.35 ± 15.39d	Ischemic	rTMS	Conventional rehab	①
Galvão 2014 (55)	T:10 C:10	T:57.4 ± 12.0/C:64.6 ± 6.8	T:47.8 ± 43.2d/C:58.9 ± 27.2d	Both	rTMS	Conventional rehab	①
Chen 2021 (56)	T1:25	T1:56.00 ± 15.56/C:63.33 ± 15.56	T1:7.33 ± 3.70d/C:6.17 ± 4.07d	Ischemic	rTMS	Sham stimulation + Conventional rehab	①②
	T2:25	T2:59.33 ± 13.33	T2:6.00 ± 3.70d				
	T3:25	T3:57.67 ± 17.78	T3:7.67 ± 5.19d				
Chen 2020 (57)	T1:19	T1:52.56 ± 11.65	Subacute	Ischemic	rPMS	Conventional rehab	①②
	T2:16	T2:48.68 ± 14.11			rTMS		
Fujimura 2024 (58)	T:22 C:24	69 ± 13/61 ± 15	34 ± 23d/41 ± 20d	Both	rPMS	Conventional rehab	①
Ke 2022 (59)	T:13 C:14	55.08 ± 13.70/54.54 ± 12.45	23.02 ± 28.65d/13.99 ± 14.53d	Hemorrhagic	rPMS	Conventional rehab	①
Kim 2020 (60)	T:40 C:37	61.2 ± 11.2/62.9 ± 13.1	14 ± 9d/16 ± 15d	Ischemic	rTMS	Conventional rehab	①②
Li 2016 (61)	T1:43 C:42	54.00 ± 13.35/53.13 ± 13.72	1.36 ± 1.48 m/1.58 ± 1.52 m	Ischemic	rTMS	Sham stimulation	①
	T2:42	57.87 ± 12.89	1.86 ± 1.12 m		rTMS		
Li 2025 (62)	T1:33 C:33	64.2 ± 10.6/64.3 ± 8.5	18.23 ± 7.63d/20.13 ± 4.74d	Ischemic	rTMS	Conventional rehab	①②
	T2:22	59.8 ± 10.0	16.63 ± 9.41d				
Liang 2024 (63)	T1:15 C:15	56.6 ± 9.1y/54.3 ± 7.6y	2.3 ± 1.8 m/2.5 ± 1.4 m	Both	rPMS + rTMS	Conventional rehab	①②
	T2:15	54.1 ± 9.6y	2.3 ± 1.5 m		rTMS		
Long 2018 (64)	T1:21 C:21	55.90 ± 8.89/56.85 ± 5.48	19.81 ± 2.98d/19.05 ± 2.74d	Both	rTMS	Conventional rehab	①
	T2:21	57.00 ± 11.78	19.57 ± 2.34d		rTMS		
Luk 2022 (65)	T:12 C:12	67.3 ± 5.8/65.1 ± 3.1	13.6 ± 6.1 w/15.1 ± 7.0 w	Ischemic	rTMS	Conventional rehab	①
Motamed 2014 (66)	T:6 C:6	55.17 ± 5.42/57.00 ± 8.67	24.00 ± 8.29 m/23.00 ± 8.94 m	Both	rTMS	Conventional rehab	①②
Obayashi 2020 (67)	T:10 C:9	64.3 ± 13.1/72.3 ± 10.7	9.2 ± 4.4 m/5.8 ± 2.2 m	Both	rPMS	Conventional rehab	①
Qin 2023 (68)	T1:15 C:14	55.87 ± 10.50/59.43 ± 9.12	3.20 ± 1.93 m/2.85 ± 1.74 m	Ischemic	rTMS	Conventional rehab	①②
	T2:20	60.05 ± 9.97	3.45 ± 1.76 m		rTMS + rPMS		
Sharma 2020 (69)	T:47 C:49	54.85 ± 13.39/52.89 ± 14.95	77.51 ± 5.38d/77.34 ± 10.21d	Ischemic	rTMS	Conventional rehab	①②
Wu 2023 (70)	T1:15 C:15	54.60 ± 11.16/55.33 ± 10.30	38.50 ± 6.36d/39.25 ± 15.31d	Both	rTMS + rPMS	Sham stimulation	①②
	T2:15	57.00 ± 10.76	33.50 ± 7.51d		rTMS		
	T3:15	54.87 ± 11.60	33.00 ± 11.69d		rPMS		
Xu 2025 (71)	T1:15	55.80 ± 14.0	48.73 ± 6.13d	Both	rTMS	Conventional rehab	①②
	T2:15	53.60 ± 16.45	45.09 ± 7.20d		rPMS		
	T3:16	56.40 ± 13.83	47.83 ± 8.26d		rTMS + rPMS		
Yang 2021 (72)	T:12 C:13	64 ± 8/64 ± 8	64 ± 23d/75 ± 49d	Both	rTMS + Conventional rehab	Conventional rehab	①②
Ji 2014 (73)	T:16 C:16	54.62 ± 5.24/57.06 ± 7.35	7.81 ± 2.43 m/7.93 ± 2.76 m	Both	rTMS	Conventional rehab	①
Fawaz 2023 (74)	T:40 C:40	57.33 ± 10.67	6w-60w	Both	rPMS	Conventional rehab	①

① Upper limb motor function; ② Activities of Daily Living (ADL).

## 3 Results

### 3.1 Literature search process

A total of 5,655 records were initially identified through database searches. After removal of duplicates, 4,635 records remained. Based on screening of titles and abstracts, 4,509 irrelevant records were excluded. The full texts of the remaining 126 articles were assessed, of which 78 were excluded for reasons including review or conference publication type, ineligible interventions, unavailable or non-extractable data, duplicate publication, ineligible outcome measures, or inability to obtain the full text. Finally, 48 randomized controlled trials (27–74) were included in the meta-analysis. The study selection process is shown in Figure 1.

**Figure 1:**
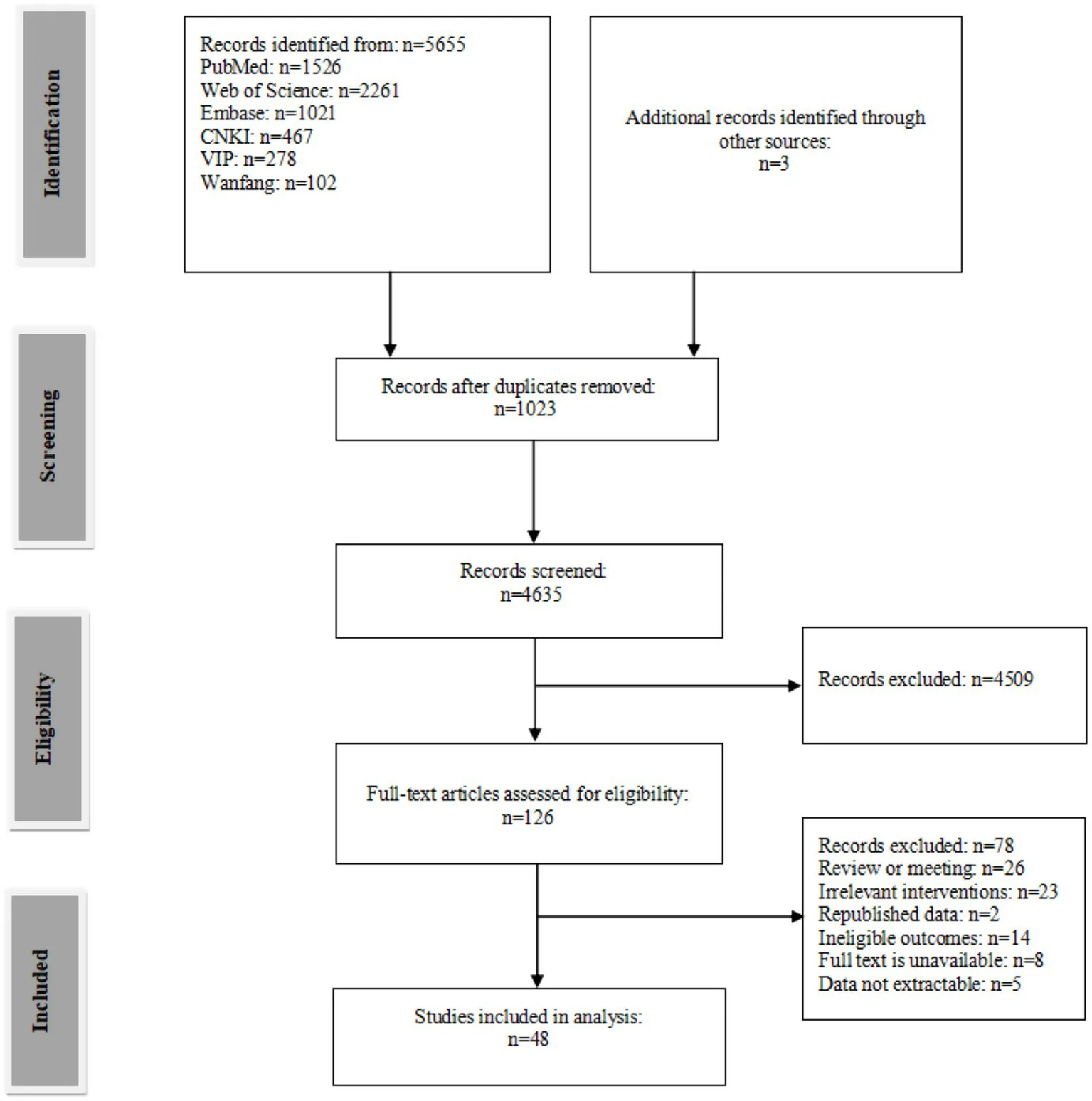
Flowchart of literature screening.

### 3.2 Methodological quality assessment

A total of 48 studies were included in this review, and their methodological quality was assessed using RoB 2. As shown in Figure 2, the RoB 2 assessment showed that, among the 48 studies, 25 studies (52.1%) were judged to have a low risk of bias, 18 studies (37.5%) had some concerns, and 5 studies (10.4%) were judged to have a high risk of bias. Overall, most studies were rated as having a low risk of bias or some concerns. The main sources of bias were concentrated in the domains of deviations from intended interventions and selection of the reported result.

**Figure 2:**
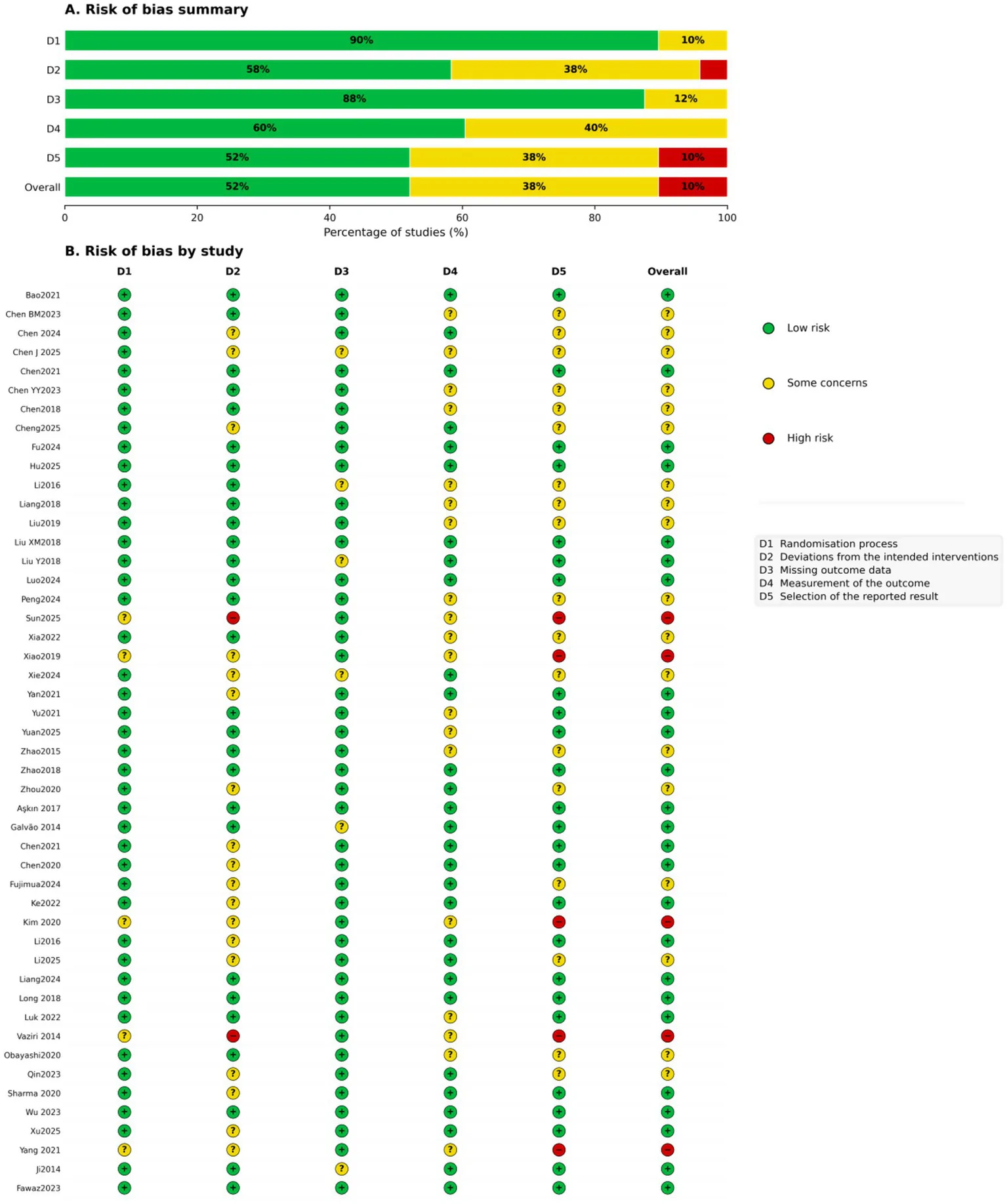
Risk-of-bias assessment using RoB 2. **(A)** Summary of risk-of-bias judgments across studies; **(B)** Risk-of-bias judgments for individual studies.

### 3.3 Basic characteristics of the included studies

A total of 48 studies were finally included, with publication years ranging from 2014 to 2025. The sample size of individual studies ranged from 12 to 180 participants. The mean age of participants was mainly between 50 and 70 years. Regarding stroke type, 19 studies included only patients with ischemic stroke, 2 studies included only patients with hemorrhagic stroke, and the remaining 27 studies included patients with both types of stroke. Disease duration ranged from several days after stroke onset, representing the acute stage, to several years after stroke, representing the chronic stage. In terms of interventions, the magnetic stimulation modalities used in the experimental groups mainly included rTMS, rPMS, and combined MS. The control groups mainly received sham stimulation combined with conventional rehabilitation training or conventional rehabilitation training alone. The basic characteristics of the included studies are presented in Table 1. Detailed magnetic stimulation intervention strategies are presented in Supplementary Material 2.

### 3.4 Network meta-analysis

#### 3.4.1 Heterogeneity and inconsistency tests

The heterogeneity analysis showed substantial heterogeneity in the overall network for upper limb motor function (τ = 0.788, τ^2^ = 0.621, Q = 320.68, df = 58, *p* < 0.001, I^2^ = 81.9%). For the ADL outcome, moderate to substantial heterogeneity was observed in the overall network (τ = 0.424, τ^2^ = 0.179, Q = 116.79, df = 43, *p* < 0.001, I^2^ = 63.2%). The global inconsistency test for upper limb motor function did not detect significant inconsistency (χ^2^ = 3.85, df = 8, *p* = 0.870), and local node-splitting analyses showed no statistically significant differences between direct and indirect evidence for the main comparisons (*p* > 0.05). Similarly, the global inconsistency test for ADL did not identify significant inconsistency (χ^2^ = 5.71, df = 13, *p* = 0.956), and local node-splitting analyses showed no statistically significant differences between direct and indirect evidence for the main comparisons (*p* > 0.05). Therefore, a random-effects consistency model was used for the network meta-analysis.

#### 3.4.2 Results of the network meta-analysis

The evidence network plots for each outcome are shown in Figure 3. In the plots, nodes represent different interventions, and the node size indicates the number of participants included for each intervention. Lines between nodes indicate direct comparisons between interventions, with thicker lines representing a larger number of included studies.

**Figure 3:**
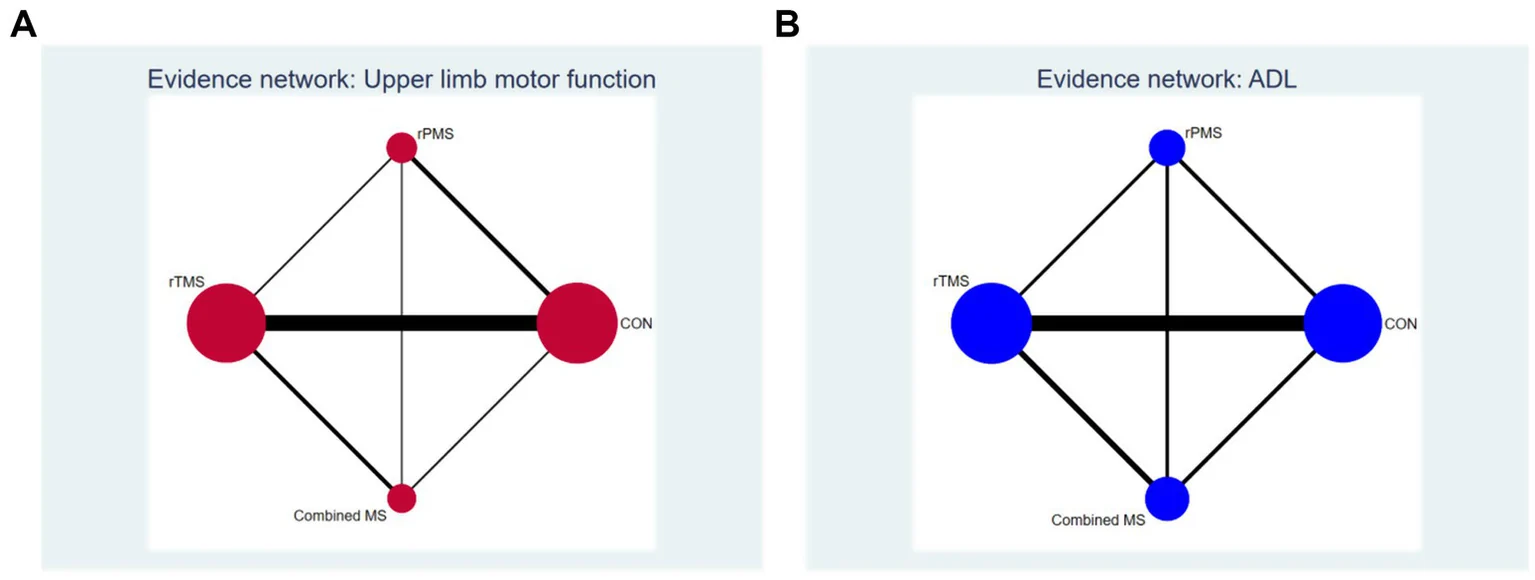
Network evidence plot. **(A)** Upper limb motor function; **(B)** ADL.

For upper limb motor function, 48 studies were included. The SMD was used as the effect size, and 95% CIs were used for statistical inference. The network meta-analysis showed that, compared with conventional rehabilitation therapy, rPMS (SMD = 0.65, 95% CI: 0.19 to 1.12), rTMS (SMD = 0.96, 95% CI: 0.68 to 1.24), and combined MS (SMD = 1.01, 95% CI: 0.47 to 1.55) significantly improved upper limb motor function. Comparisons among the magnetic stimulation interventions did not reach statistical significance (*p* > 0.05). The SUCRA ranking results showed that combined MS ranked highest (SUCRA = 81.3%, PrBest = 55.8%, MeanRank = 1.6), followed by rTMS (SUCRA = 77.0%, PrBest = 39.0%, MeanRank = 1.7), rPMS (SUCRA = 41.6%, PrBest = 5.2%, MeanRank = 2.8), and conventional rehabilitation therapy (SUCRA = 0.1%, PrBest = 0.0%, MeanRank = 4.0). These findings suggest that rTMS combined with rPMS and rTMS had relatively high ranking probabilities. However, because the differences between these two interventions and other active interventions were not statistically significant, the ranking results should be interpreted cautiously in conjunction with the effect estimate and 95% CI (Table 2).

**Table 2:** League table of the effects of different MS interventions on upper limb motor function in patients with stroke.

Treatment	CON	rPMS	rTMS	Combined MS
CON	–			
rPMS	0.655 (0.194, 1.116)	–		
rTMS	0.960 (0.679, 1.241)	0.305 (−0.192, 0.802)	–	
Combined MS	1.011 (0.467, 1.555)	0.356 (−0.287, 0.998)	0.051 (−0.469, 0.571)	–

For ADL, 32 studies were included. The SMD was used as the effect size, and 95% CIs were used for statistical inference. The network meta-analysis showed that, compared with conventional rehabilitation therapy, rPMS (SMD = 0.60, 95% CI: 0.26 to 0.95), rTMS (SMD = 0.76, 95% CI: 0.56 to 0.97), and combined MS (SMD = 1.27, 95% CI: 0.94 to 1.59) significantly improved ADL. Comparisons among magnetic stimulation interventions showed no statistically significant difference between rTMS and rPMS (SMD = 0.16, 95% CI: −0.19 to 0.51). Combined MS was superior to rPMS (SMD = 0.66, 95% CI: 0.26 to 1.07) and rTMS (SMD = 0.50, 95% CI: 0.18 to 0.82), with statistically significant differences. The SUCRA ranking results showed that combined MS ranked highest (SUCRA = 99.9%, PrBest = 99.8%, MeanRank = 1.0), followed by rTMS (SUCRA = 60.7%, PrBest = 0.1%, MeanRank = 2.2), rPMS (SUCRA = 39.3%, PrBest = 0.0%, MeanRank = 2.8), and conventional rehabilitation therapy (SUCRA = 0.0%, PrBest = 0.0%, MeanRank = 4.0). These results suggest that rTMS combined with rPMS may have a higher probability of being a favorable intervention for improving ADL (Table 3).

**Table 3:** League table of the effects of different MS interventions on ADL in patients with stroke.

Treatment	CON	rPMS	rTMS	Combined MS
CON	–			
rPMS	0.603 (0.260, 0.945)	–		
rTMS	0.765 (0.558, 0.972)	0.162 (−0.190, 0.513)	–	
Combined MS	1.265 (0.937, 1.593)	0.663 (0.259, 1.066)	0.501 (0.179, 0.822)	–

### 3.5 Sensitivity analysis

To examine the robustness of the findings, a sensitivity analysis was conducted for the ADL outcome. After excluding studies that reported only the BI, only studies using the MBI were retained for repeated network meta-analysis, to evaluate the influence of combining BI and MBI on the results. The sensitivity analysis showed that, compared with conventional rehabilitation therapy, rPMS (SMD = 0.51, 95% CI: 0.15 to 0.88, *p* = 0.006), rTMS (SMD = 0.71, 95% CI: 0.50 to 0.92, *p* < 0.001), and combined MS (SMD = 1.27, 95% CI: 0.94 to 1.60, *p* < 0.001) still significantly improved ADL. Comparisons among magnetic stimulation interventions showed no statistically significant difference between rTMS and rPMS (SMD = 0.19, 95% CI: −0.18 to 0.57, *p* = 0.308). Combined MS remained superior to rPMS (SMD = 0.76, 95% CI: 0.35 to 1.17, *p* < 0.001) and rTMS (SMD = 0.56, 95% CI: 0.24 to 0.89, *p* < 0.001), with statistically significant differences. The SUCRA ranking results showed that combined MS ranked highest (SUCRA = 100.0%, PrBest = 99.9%, MeanRank = 1.0), followed by rTMS (SUCRA = 61.6%, PrBest = 0.0%, MeanRank = 2.2), rPMS (SUCRA = 38.3%, PrBest = 0.0%, MeanRank = 2.9), and conventional rehabilitation therapy (SUCRA = 0.1%, PrBest = 0.0%, MeanRank = 4.0). Overall, after excluding studies that reported only the BI, the main effect directions, statistical significance, and intervention ranking for the ADL outcome were largely consistent with those of the primary analysis, suggesting that combining BI and MBI did not materially alter the main conclusions (Table 4). A sensitivity analysis excluding studies at high risk of bias was not performed because evidence for some treatment comparisons was limited, and excluding the five high-risk studies would further reduce the available evidence for these comparisons. Consequently, the robustness of the pooled estimates and treatment rankings to the exclusion of high-risk studies remains uncertain. Risk of bias was considered in the RoB 2 assessment, GRADE evaluation, and interpretation of the findings.

**Table 4:** League table of the effects of different magnetic stimulation interventions on ADL after excluding studies that reported only the Barthel Index.

Treatment	CON	rPMS	rTMS	Combined MS
CON	–			
rPMS	0.512 (0.147, 0.877)	–		
rTMS	0.707 (0.499, 0.915)	0.195 (−0.180, 0.569)	–	
Combined MS	1.272 (0.941, 1.602)	0.759 (0.348, 1.171)	0.564 (0.240, 0.889)	–

### 3.6 Publication bias

Comparison-adjusted funnel plots and Egger’s test were used to assess publication bias and small-study effects (Figure 4). For upper limb motor function, the comparison-adjusted funnel plot showed that the study points were generally distributed around the center line, without obvious asymmetry. Egger’s test also did not indicate significant funnel plot asymmetry (*t* = 1.09, *p* = 0.279). For ADL, the comparison-adjusted funnel plot likewise showed no obvious asymmetric distribution, and Egger’s test was not statistically significant (*t* = −0.40, *p* = 0.688). Therefore, no clear evidence of publication bias or small-study effects was identified for either outcome.

**Figure 4:**
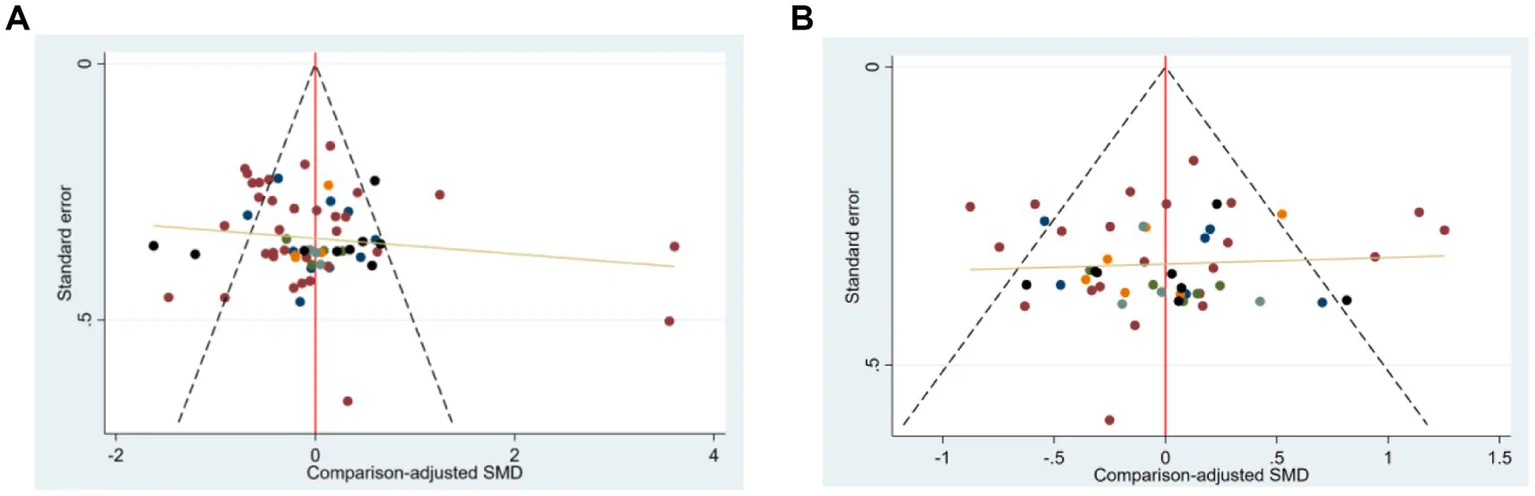
Funnel plots for publication bias assessment. **(A)** Upper limb motor function; **(B)** ADL.

### 3.7 Certainty of evidence

The GRADE assessment of the certainty of evidence showed that the effects of different MS interventions on upper limb motor function and ADL in patients with stroke were supported by low-certainty evidence (Figure 5). The certainty of evidence was mainly downgraded because of insufficient implementation of blinding in the included studies and substantial heterogeneity across studies.

**Figure 5:**
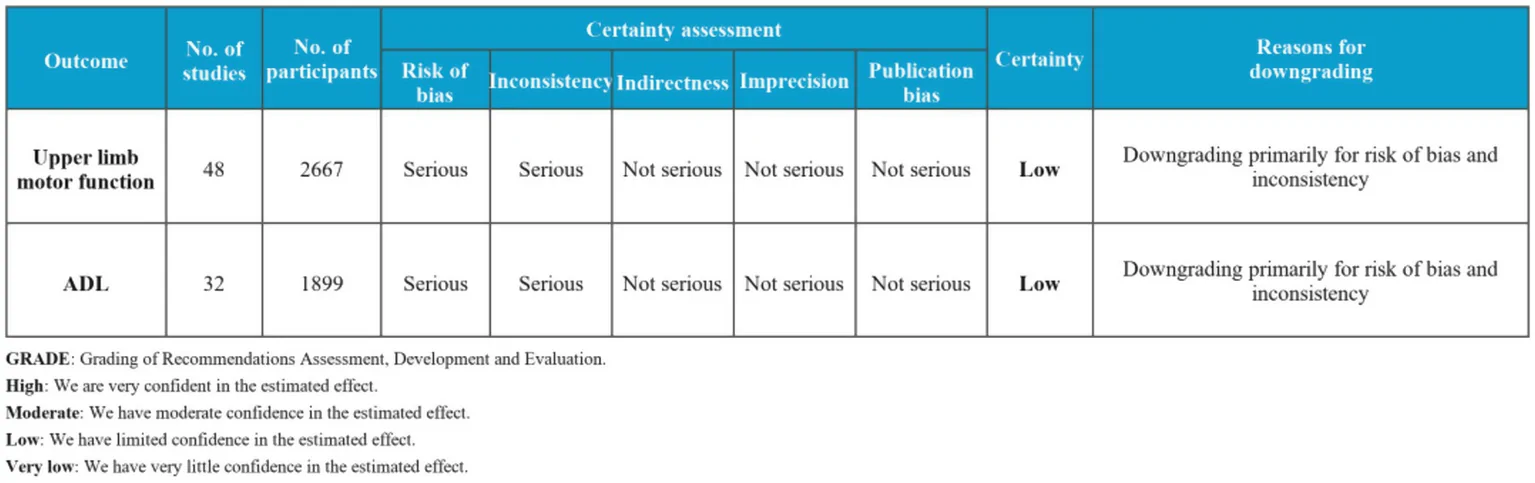
GRADE assessment of the certainty of evidence.

## 4 Discussion

In this network meta-analysis, we systematically evaluated the rehabilitative effects of different magnetic stimulation modalities on upper limb motor function and ADL in patients with stroke. The results showed that rTMS, rPMS, and combined magnetic stimulation were all significantly superior to conventional rehabilitation therapy in improving upper limb motor function and ADL. These findings further support the potential value of magnetic stimulation in post-stroke rehabilitation and are generally consistent with previous studies (14, 16, 75, 76). For upper limb motor function, although pairwise comparisons among the three magnetic stimulation modalities did not reach statistical significance, the SUCRA ranking results showed that combined MS and rTMS had relatively high ranking probabilities, with combined MS ranking slightly higher (SUCRA = 81.3%), followed by rTMS (SUCRA = 77.0%). These findings suggest that combined magnetic stimulation and rTMS may be favorable intervention options for improving upper limb motor function. For ADL, combined MS not only had the highest SUCRA value (SUCRA = 99.9%) but was also significantly superior to rTMS and rPMS alone in pairwise comparisons, suggesting that combined magnetic stimulation may have a more apparent advantage in improving ADL. The GRADE assessment indicated that the certainty of evidence for both upper limb motor function and ADL was low, mainly because of risk of bias and substantial between-study heterogeneity.

It should be noted that clinical heterogeneity among the included studies may have influenced the estimation of treatment effects, treatment rankings, and the stability of SUCRA values. Stroke stage may affect the therapeutic response to magnetic stimulation. Previous studies have suggested that there may be sensitive time windows after stroke during which patients show greater responsiveness to rehabilitation training. In the early and subacute stages, neuroplasticity and spontaneous recovery processes are relatively active, and patients may therefore be more likely to benefit from neuromodulation and rehabilitation training. In contrast, patients in the chronic stage may have different recovery potential and treatment response patterns (77–79). In addition, ischemic and hemorrhagic stroke differ in injury mechanisms, lesion characteristics, and recovery processes (80, 81). The trajectories of ADL and functional recovery may therefore not be identical across stroke types, and the same magnetic stimulation protocol may produce different therapeutic effects. Stimulation parameters also jointly determine the direction of neuromodulation and the dose–response relationship, and may directly influence the magnitude of the effect estimate. Therefore, SUCRA rankings should be interpreted as probabilistic rankings based on the currently available mixed evidence, rather than as definitive evidence of superiority across all stroke types, disease stages, and stimulation parameters. These variables were also considered as potential effect modifiers when assessing the transitivity assumption. Although their distributions appeared clinically acceptable for indirect comparisons, incomplete and inconsistent reporting across primary studies means that some residual imbalance cannot be excluded.

For upper limb motor function, this study found that combined MS and rTMS had relatively high ranking probabilities. The FMA-UE has good reliability, validity, and sensitivity for assessing upper limb motor function in patients with stroke. From a neurophysiological perspective, one of the core pathophysiological mechanisms underlying post-stroke upper limb motor dysfunction is an imbalance in interhemispheric inhibition (82). Both high-frequency rTMS applied to the ipsilesional M1 and low-frequency rTMS applied to the contralesional M1 can modulate cortical excitability in a frequency-dependent manner and help restore the dynamic balance between the two hemispheres. Previous evidence has shown that high-frequency rTMS over the ipsilesional M1 and low-frequency rTMS over the contralesional M1 are both superior to sham stimulation or conventional rehabilitation alone in improving upper limb motor function. The study by Peng et al. (43) further demonstrated that low-frequency rTMS can inhibit excessive activation of the unaffected hemisphere, reshape the balance of interhemispheric excitability, and significantly improve FMA-UE scores. Xiao et al. (46) showed that high-frequency rTMS can directly enhance excitability in the lesioned hemisphere and similarly promote upper limb motor recovery. Additional evidence suggests that rTMS has unique advantages in inducing neuroplastic changes. Several studies (83–85) have found that rTMS can significantly increase motor evoked potential amplitudes, indicating enhanced corticospinal excitability, and can induce synaptic effects resembling long-term potentiation or long-term depression. These mechanisms may explain why rTMS showed relatively large effect estimates and high ranking probabilities in this study.

Combined MS showed a slightly higher SUCRA ranking than rTMS for upper limb motor function, which may be related to its simultaneous effects on both the central and peripheral nervous systems. rTMS mainly acts by modulating motor cortical excitability, improving interhemispheric inhibition imbalance, and promoting recovery of corticospinal pathway function (86). rPMS, in contrast, can stimulate peripheral neuromuscular structures, induce muscle contraction, and enhance proprioceptive input and sensorimotor feedback from the affected limb (87). Because post-stroke upper limb motor recovery depends not only on central motor output but also on peripheral sensory feedback, muscle recruitment, and reconstruction of motor patterns, combined MS may promote upper limb motor recovery through the synergistic effects of central modulation and peripheral input (71, 88). However, combined-MS protocols were not completely uniform across studies, because the sequence of rTMS and rPMS, stimulation intensity, frequency, treatment duration, and total dose differed among trials. This within-category variability should be considered when interpreting the pooled effect of combined MS.

This study also found that, for ADL assessed using BI/MBI, combined magnetic stimulation had the highest SUCRA value and was significantly more effective than rTMS or rPMS alone. Unlike the FMA-UE, which focuses more on upper limb motor control, ADL reflects patients’ integrated functional performance in daily tasks such as eating, dressing, transferring, and personal hygiene. It depends not only on improvements in muscle strength and range of motion but also on multisystem coordination involving motor initiation, postural control, integration of sensory feedback, bilateral limb coordination, and task execution (89). Therefore, combined magnetic stimulation, which simultaneously modulates central motor networks and enhances peripheral sensorimotor input, may be more consistent with the characteristics of “global functional recovery” reflected by ADL. This finding is also compatible with the concept of a “central-peripheral-central” closed-loop rehabilitation model (90). Central intervention may regulate functional network activity and synaptic plasticity in relevant brain regions, thereby influencing the generation and transmission of motor commands. Peripheral intervention may enhance standardized movement patterns and proprioceptive input, feed peripheral sensorimotor information back to the central nervous system, and participate in functional integration between the sensory and motor cortices (91). This closed-loop regulatory mechanism may help explain the more apparent advantage of combined magnetic stimulation for ADL outcomes. However, the present study is a synthesis of clinical outcome evidence, and the underlying mechanisms require further validation in future basic and clinical mechanistic studies.

In addition, although combined magnetic stimulation showed potential advantages in improving upper limb motor function and ADL, its implementation in real-world rehabilitation settings requires consideration of accessibility, cost, and feasibility. Combined protocols usually require two types of magnetic stimulation equipment and place higher demands on therapists’ operational skills, treatment workflow arrangements, and site resources. In primary care or resource-limited rehabilitation institutions, its application may be constrained by equipment availability and service costs. Therefore, future studies should not only further verify its clinical efficacy but also conduct cost-effectiveness analyses and real-world studies to clarify the target populations and feasibility of combined magnetic stimulation in healthcare settings with different resource levels.

### 4.1 Limitations

This study focused on the effects of stimulation frequency and site in conventional rTMS. Although TBS also belongs to the category of magnetic stimulation, it uses a high-frequency burst stimulation pattern and differs substantially from conventional rTMS in its mechanism of action and single-session treatment duration. To ensure the homogeneity of interventions in the network meta-analysis, TBS was not included in this study. Future research may further compare the efficacy of conventional rTMS and TBS in stroke rehabilitation. Heterogeneity existed among the included studies in stimulation parameters, such as frequency, intensity, site, and treatment duration, as well as in patient characteristics, such as stroke type, disease duration, and disease severity, which may affect the stability of the results. Although SUCRA rankings suggested relative ranking trends, the direct comparisons for upper limb motor function showed no statistically significant differences among active magnetic stimulation interventions. This may be partly due to the limited number and sample size of studies on rPMS and combined magnetic stimulation. Although the comparison-adjusted funnel plots and Egger’s tests did not indicate obvious publication bias, the possibility of publication bias or small-study effects cannot be completely excluded because some treatment comparisons included a limited number of studies. Therefore, the conclusions need to be validated by more large-sample studies.

## 5 Conclusion

Different magnetic stimulation interventions may have positive effects on upper limb motor function and ADL in patients with stroke. The SUCRA rankings showed that combined magnetic stimulation and rTMS had relatively high ranking probabilities for upper limb motor recovery, while combined magnetic stimulation had a relatively high ranking probability for improving ADL. However, these findings should be interpreted with caution because the included studies differed in stroke stage, stroke type, stimulation parameters, and treatment dose, some direct comparisons did not reach statistical significance, and the certainty of evidence was low. Future high-quality head-to-head trials are needed to further validate the relative efficacy of different magnetic stimulation protocols.
