# Supplementary material for: Comparative effects of different magnetic stimulation modalities on upper limb motor function and activities of daily living in patients with stroke: a systematic review and network meta-analysis
Source: Front Neurol. 2026 Oct 27;17:1842774. doi: 10.3389/fneur.2026.1842774 (PMC13747782; doi:10.3389/fneur.2026.1842774)
Supplement: Supplementary file 1 [file Table_1.docx]

Table S1 Literature search strategies

| Database |  |
| --- | --- |
| PubMed | #1 ("transcranial magnetic stimulation"[Title/Abstract] OR "repetitive transcranial magnetic stimulation"[Title/Abstract] OR "rTMS"[Title/Abstract] OR "TMS"[Title/Abstract] OR "peripheral magnetic stimulation"[Title/Abstract] OR "repetitive peripheral magnetic stimulation"[Title/Abstract] OR "rPMS"[Title/Abstract] OR "magnetic stimulation"[Title/Abstract] OR "combined magnetic stimulation"[Title/Abstract] OR "central-peripheral magnetic stimulation"[Title/Abstract])  #2 ("stroke"[Title/Abstract] OR "poststroke"[Title/Abstract] OR "post-stroke"[Title/Abstract] OR "cerebrovascular accident"[Title/Abstract] OR "CVA"[Title/Abstract] OR "brain ischemia"[Title/Abstract] OR "intracerebral hemorrhage"[Title/Abstract] OR "cerebral hemorrhage"[Title/Abstract] OR "hemiparesis"[Title/Abstract] OR "hemiplegia"[Title/Abstract])  #3 ("upper limb"[Title/Abstract] OR "upper extremity"[Title/Abstract] OR "arm"[Title/Abstract] OR "hand"[Title/Abstract] OR "Fugl-Meyer"[Title/Abstract] OR "FMA"[Title/Abstract] OR "FMA-UE"[Title/Abstract] OR "motor function"[Title/Abstract] OR "activities of daily living"[Title/Abstract] OR "ADL"[Title/Abstract] OR "Barthel Index"[Title/Abstract] OR "BI"[Title/Abstract] OR "Modified Barthel Index"[Title/Abstract] OR "MBI"[Title/Abstract])  #4 Randomized controlled trial [Publication Type]  #5 #1 AND #2 AND #3 AND #4 |
| Web of Science | #1 TS=("transcranial magnetic stimulation" OR "repetitive transcranial magnetic stimulation" OR "rTMS" OR "TMS" OR "peripheral magnetic stimulation" OR "repetitive peripheral magnetic stimulation" OR "rPMS" OR "magnetic stimulation" OR "combined magnetic stimulation" OR "central-peripheral magnetic stimulation")  #2 TS=("stroke" OR "poststroke" OR "post-stroke" OR "cerebrovascular accident" OR "CVA" OR "brain ischemia" OR "intracerebral hemorrhage" OR "cerebral hemorrhage" OR "hemiparesis" OR "hemiplegia")  #3 TS=("upper limb" OR "upper extremity" OR "arm" OR "hand" OR "Fugl-Meyer" OR "FMA" OR "FMA-UE" OR "motor function" OR "activities of daily living" OR "ADL" OR "Barthel Index" OR "BI" OR "Modified Barthel Index" OR "MBI")  #4 TS=("Randomized controlled trial" OR "RCT" OR "randomized" OR "randomised")  #5 #1 AND #2 AND #3 AND #4 |
| EMbase | #1 'transcranial magnetic stimulation':ab,ti OR 'repetitive transcranial magnetic stimulation':ab,ti OR 'rtms':ab,ti OR 'tms':ab,ti OR 'peripheral magnetic stimulation':ab,ti OR 'repetitive peripheral magnetic stimulation':ab,ti OR 'rpms':ab,ti OR 'magnetic stimulation':ab,ti OR 'combined magnetic stimulation':ab,ti OR 'central-peripheral magnetic stimulation':ab,ti  #2 'stroke':ab,ti OR 'poststroke':ab,ti OR 'post-stroke':ab,ti OR 'cerebrovascular accident':ab,ti OR 'cva':ab,ti OR 'brain ischemia':ab,ti OR 'intracerebral hemorrhage':ab,ti OR 'cerebral hemorrhage':ab,ti OR 'hemiparesis':ab,ti OR 'hemiplegia':ab,ti  #3 'upper limb':ab,ti OR 'upper extremity':ab,ti OR 'arm':ab,ti OR 'hand':ab,ti OR 'fugl-meyer':ab,ti OR 'fma':ab,ti OR 'fma-ue':ab,ti OR 'motor function':ab,ti OR 'activities of daily living':ab,ti OR 'adl':ab,ti OR 'barthel index':ab,ti OR 'bi':ab,ti OR 'modified barthel index':ab,ti OR 'mbi':ab,ti  #4 'randomized controlled trial':ab,ti OR 'rct':ab,ti OR 'randomized':ab,ti OR 'randomised':ab,ti  #5 #1 AND #2 AND #3 AND #4 |
| CNKI | (磁刺激 + 重复经颅磁刺激 + 外周磁刺激 + 重复外周磁刺激 + 功能性磁刺激 + 非侵入性脑刺激)ADN（脑卒中 + 中风 + 偏瘫 + 脑出血 + 脑梗死）ADN(上肢 + 上肢运动功能 + 肢体功能 + 日常生活活动能力+ 日常活动能力) |
| Wngfang、VIP | (磁刺激 OR 重复经颅磁刺激 OR 外周磁刺激 OR 重复外周磁刺激 OR 功能性磁刺激 OR 非侵入性脑刺激)ADN（脑卒中 OR 中风 OR 偏瘫 OR 脑出血 OR 脑梗死）ADN(上肢 OR 上肢运动功能 OR 肢体功能 OR 日常生活活动能力OR 日常活动能力) |
